# Health-Related Quality of Life and Perceived Stigma in Eosinophilic Esophagitis: A Real-World, US, Web-Based Survey

**DOI:** 10.1016/j.gastha.2024.07.015

**Published:** 2024-07-30

**Authors:** Benjamin D. Gold, Bridgett Goodwin, Kimberly Davis, Carolyn Sweeney, Maria Reynolds, Jeanne Jiang, Tao Fan, Mena Boules, Szu-Ta Chen, David A. Katzka

**Affiliations:** 1 GI Care for Kids, Children’s Center for Digestive Healthcare, LLC, Atlanta, Georgia; 2 Takeda Development Center Americas, Inc, Cambridge, Massachusetts; 3 RTI Health Solutions, Research Triangle Park, North Carolina; 4 Takeda Pharmaceuticals USA, Inc, Lexington, Massachusetts; 5 Division of Digestive and Liver Diseases, New York-Presbyterian/Columbia University Irving Medical Center, New York City, New York

**Keywords:** Eosinophilic Esophagitis, Health-Related Quality of Life, Anxiety, Depression, Perceived Stigma

## Abstract

**Background and Aims:**

Eosinophilic esophagitis (EoE) is associated with impaired health-related quality of life (HRQoL) and stigma perceptions. Therefore, we examined the real-world impact of EoE on the daily life and ability to function in adolescents (caregiver-reported) and adults with EoE in the United States of America in a noninterventional, cross-sectional, web-based survey.

**Methods:**

HRQoL was assessed using the Short Form Health Survey (domains: vitality and social functioning) and the European Health Interview Survey (domain: sleep). Scores for the survey responses were on a scale of 0 to 100; higher scores indicated better performance in the HRQoL domain. Anxiety and depression were assessed using the Patient-Reported Outcomes Measurement Information System (PROMIS) short forms. Higher PROMIS scores indicated higher levels of anxiety and depression; a mean score of 50.0 was representative of the general population (individuals without EoE). The sources and impact of EoE-associated perceived stigma were also examined.

**Results:**

Overall, 211 caregivers and 184 adults completed the survey. HRQoL scores were slightly higher for adolescents than adults with EoE (adolescent and adult scores, respectively: vitality, 50.3 and 36.1; social functioning, 64.0 and 62.4; and sleep, 55.7 and 52.0). Anxiety scores (adolescent and adult scores, respectively: 54.8 and 59.7) and depression scores (54.5 and 56.3) were higher in those with EoE than in the general population. Most participants reported experiencing perceived stigma, which was most commonly from family, friends, classmates, or health-care professionals.

**Conclusion:**

Patients with EoE had poor HRQoL, which was demonstrated by the high EoE-associated emotional and psychological burdens and perceived stigma they experience.

## Graphical abstract

**Figure:**
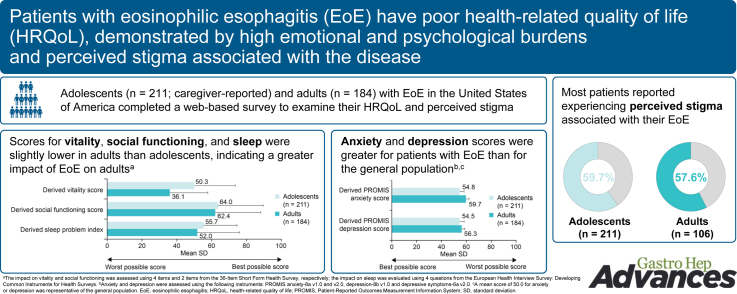

## Introduction

Eosinophilic esophagitis (EoE) is an immune-mediated disorder characterized by esophageal dysfunction and chronic mucosal eosinophilia.^1^ Although clinical symptoms of EoE can vary with age, adolescents and adults often report dysphagia and food impaction.^1^

Obtaining an EoE diagnosis may require visits to multiple health-care professionals (HCPs),^2^ often due to poor disease awareness,^3^ nonspecific symptom presentation and symptoms getting overlooked because of patients’ adaptive eating strategies.^4^ Consequently, patients can experience diagnostic delays of up to 8 years from symptom onset.^5^

Current guidelines to manage EoE recommend off-label treatment with proton-pump inhibitors, swallowed topical corticosteroids, or dietary elimination.^6^ Monitoring disease progression and treatment response requires patients to undergo repeat endoscopies and esophageal biopsies.^7^ To maintain disease remission, long-term pharmacologic treatments^8^ or dietary exclusions^9^ are recommended.^8^^,^^9^ Repeated esophageal dilations are suggested to provide symptom relief to those patients with EoE and fibrostenosis.^10^

EoE is associated with impaired health-related quality of life (HRQoL)^11^ and can negatively affect sleep, leisure, school/work activities,^2^^,^^12^ social interactions, and eating behaviors, including causing concerns about choking episodes, especially in public settings.^2^^,^^13^ EoE may increase the risk of anxiety and depression, owing to the burden associated with the disease and management strategies.^14^ Furthermore, patients with EoE are susceptible to perceived stigma,^15^ which may be attributed to associated eating behaviors such as avoidant restrictive food intake disorder.^16^ Perceived stigma can worsen the impact of other chronic gastrointestinal diseases, such as eosinophilic gastrointestinal disorders (EGID)^15^ and inflammatory bowel disease (IBD),^17^ leading to poor HRQoL,^15^^,^^17^ anxiety and depression,^15^^,^^17^ high health-care resource utilization,^15^ and suboptimal therapy compliance.^17^

This study was a comprehensive analysis of the real-world impact of EoE on daily life and the ability to function in a nationwide population of adolescents and adults in the United States of America. In particular, this study focused on physical and social functioning, emotional and psychological consequences, and perceived stigma in patients with EoE.

## Methods

### Study Design and Participants

This noninterventional, cross-sectional, web-based survey included adolescents (aged 11–17 years) and adults (aged ≥18 years) with EoE. Caregivers completed the survey on behalf of adolescents. Participants were identified and recruited through the nonprofit organization Campaign Urging Research for Eosinophilic Disease (CURED), based in the United States of America.^18^ CURED advocates for patients with EGID and their families, focusing on supporting research activities and enhancing awareness of EGID.^18^ CURED members were invited to participate via e-mail and a private Facebook group, both of which included a link to a generic screening survey. Eligible participants, who were identified during screening (Figure A1), provided informed consent before study enrollment. The planned data collection period was 8 weeks or until the maximum targeted sample size of 300 completed surveys (a minimum of ≥50 surveys from caregivers and ≥100 surveys from adults) was achieved. Participants completed the survey between February 2, 2021 and February 22, 2021. Survey items examined EoE symptoms, the impact of EoE on daily life and ability to function (vitality, social functioning, sleep, and impairment), levels of anxiety and depression within this population, and stigma perceptions. Specific domains from general questionnaires that were thought to best capture HRQoL were chosen for this survey to avoid repetition with other questionnaires and to reduce the burden on the users. Upon completion of the survey, participants who provided their e-mail address received a US$40 e-gift card to show appreciation for their time.

#### Inclusion criteria

Study eligibility required a caregiver-reported (adolescents) or a self-reported (adults) physician diagnosis of EoE. Caregivers and adults with EoE (aged ≥18 years) were residents in the United States of America, were able to provide informed consent, and confirmed that they were able to read and respond to a web-based survey in English. All information provided by the participants was kept confidential and the anonymized analysis data set that was made available to the study sponsor did not include any participant information. Data collected from the survey are reported descriptively, and no statistical comparisons were performed. Caregivers of adolescents and adults with EoE are hereafter referred to as “participants”.

### Current Symptoms of EoE and Use of Prescription Medications for EoE

Participants were asked to report any symptoms of EoE being experienced at the time of the survey (current symptoms) from a prespecified list, which was developed after screening the existing literature. Participants could select more than one response and report any symptoms not listed (an open-ended response option was provided). Another question asked participants to rate their symptoms as “none”, “mild” (minimal limitation on daily activities), “moderate” (able to perform most activities but with some limitations), or “severe” (unable to perform most daily activities). Participants were also asked to report the duration between the onset of their symptoms and completing this survey, as well as how well their EoE had been controlled during the 3 months before the survey completion and whether they were taking any prescription medications for EoE at the time of the study.

### Impact of EoE on Functioning

#### Impact of EoE on vitality

The vitality domain (4 items) from the Research and Development (RAND) 36-Item Short Form Health Survey (SF-36) was used to assess the impact of EoE on participants’ vitality over the 4 weeks before survey completion.^19^ Participants were asked 4 questions related to their energy levels and the frequency of fatigue. Response options for each item were scored from 1 (all the time) to 6 (none of the time). The RAND Health (2017) scoring algorithm^20^ was used to transform scores and calculate overall vitality scores ranging from 0 to 100, where higher scores indicated greater vitality (Table A1).

#### Impact of EoE on social functioning

The social functioning domain (2 items) from RAND SF-36 was used to assess the impact of EoE on participants’ social functioning over the 4 weeks before survey completion.^19^ Participants were asked to what extent and how often their symptoms of EoE interfered with their normal social activities. Response options for each item were scored from “not at all” (score 1) to “extremely” (score 5) or “all of the time” (score 1) to “none of the time” (score 6). The RAND Health, 2017 scoring algorithm^20^ was used to transform scores and calculate overall social functioning scores ranging from 0 to 100, where higher scores indicated fewer limitations and therefore greater social functioning (Table A1).

#### Impact of EoE on sleep

The impact of symptoms of EoE on participants’ ability to fall or stay asleep was assessed using the sleep problems instrument (4 items) from the European Health Interview Survey (EUROHIS): Developing Common Instruments for Health Surveys.^21^ Participants who reported having sleeping problems (item 1) were asked 3 questions related to trouble falling asleep, waking up frequently during the night, and waking up too early over the 4 weeks before survey completion.^21^ Response options were scored from 1 (all the time) to 5 (none of the time).^21^ A composite sleep scale total score/sleep problem index was derived that ranged from 0 to 100, in which higher scores indicated fewer sleep problems and therefore better sleep; this was calculated by 100 × ([{sum of the scores for the three questions/3} − 1]/4).^21^ Sleep problem indices were only calculated for participants who reported having sleep problems and responded to at least 2 of the 3 subsequent items in the survey.

#### Impact of EoE on impairment

The 3-item Sheehan Disability Scale (SDS) evaluated the extent to which a participant’s disability due to an illness or health problem interferes with school/work, social life/leisure activities, and family life/home responsibilities.^22^ Participants were asked to indicate how much their symptoms disrupted their regular activities using a visual analog scale, with response options scored from 0 (not at all) to 10 (extremely). Scores were calculated individually for each item, where a score greater than 5 indicated impairment in that area.^23^ SDS scores were calculated as the sum of the scores for all 3 items and ranged from 0 (unimpaired) to 30 (highly impaired), where higher scores indicated greater functional impairment.^23^

#### Current symptoms of anxiety and depression

The 4 Patient-Reported Outcomes Measurement Information System (PROMIS) short-form anxiety and depression instruments evaluated anxiety and depression in adolescents (parent/caregiver proxy versions) and adults over the 7 days before survey completion.^24^

— Adolescents: PROMIS anxiety-8a v2.0 and PROMIS depressive symptoms-6a v2.0.
— Adults: PROMIS anxiety-8a v1.0 and PROMIS depression-8b v1.0.

Participants were asked to answer 8 items each on anxiety and depression (or 6 items for depression in caregivers of adolescents) and responses were scored from 1 (never) to 5 (always), with higher scores indicating higher levels of anxiety or depression. For each scale score, the raw score was calculated as the sum of the individual items; however, a summary score was not calculated if a participant did not respond to all items. Using a conversion table provided by the scoring manual for each short form, the individual raw scores (sum of the individual items) were converted into T-scores with a mean of 50 and a standard deviation (SD) of 10, which also facilitated the comparison with the general population experiencing anxiety and depression, and expected standard error estimates. Higher T-scores indicated higher levels of anxiety and depression. For items that included a response of “sometimes”, “often”, or “always”, the perspectives of caregivers and adult participants on how bothersome the respective symptom had been over the past 4 weeks were captured on a scale of 1 (not at all bothersome) to 5 (very bothersome).

### Perceived Stigma

Three questions were developed to assess participants’ experiences of stigma, which was defined as feeling discriminated against or viewed negatively owing to their symptoms of EoE (Table A2). Participants who reported experiencing stigma over the past year were asked to report the sources of this stigma (eg friends, family members, HCPs, coworkers, or classmates). Participants were also asked to state the impact of the perceived stigma from a prespecified list of responses containing an open-ended response option.

## Results

### Participant Demographics

Overall, 486 participants across all 5 regions of the United States of America responded to the invitation/advertisement (caregivers [n = 267] and adults: [n = 219]; Figure A1). The anticipated sample size was exceeded, with 395 participants completing the survey (caregivers [n = 211] and adults [n = 184]; Table 1). The mean (SD) ages for adolescents and adults with EoE were 13.8 (1.9) years and 35.5 (10.1) years, respectively. Most participants were White (92.9%); 73.9% of adolescents were male, and 75.5% of adults were female.

**Table 1:** Baseline Demographics of Adolescents (Caregiver-Reported) and Adults (Self-Reported) With EoE

Demographic	Adolescents (n = 211)	Adults (n = 184)
Age, years, mean (SD)	13.8 (1.9)	35.5 (10.1)
Sex, n (%)		
Male	156 (73.9)	42 (22.8)
Female	52 (24.6)	139 (75.5)
Prefer not to answer	3 (1.4)	3 (1.6)
Race,^a^ n (%)		
African American or Black	12 (5.7)	5 (2.7)
American Indian	4 (1.9)	6 (3.3)
Asian or Pacific Islander	2 (0.9)	2 (1.1)
White	193 (91.5)	174 (94.6)
Mixed race (2 or more races)	8 (3.8)	2 (1.1)
Other^b^	2 (0.9)	0 (0.0)
Prefer not to answer	3 (1.4)	2 (1.1)
Hispanic, Latino, or Spanish origin or descent, n (%)	11 (5.2)	10 (5.4)
Employment status, n (%)		
Employed full time	NA	90 (49.5)
Employed part time	NA	27 (14.8)
Student full time or part time	NA	19 (10.4)
Not employed and not looking for employment	NA	15 (8.2)
Not employed due to disability	NA	13 (7.1)
Not employed but looking for employment	NA	9 (4.9)
Other^c^	NA	11 (6.0)
Highest education level, n (%)		
High school diploma or equivalent (GED)	NA	51 (27.9)
College degree (eg, BA, BS)	NA	91 (49.7)
Professional or graduate degree (eg, MS, PhD, MD, JD)	NA	27 (14.8)
Other^d^	NA	15 (8.2)
Region of the United States of America,^e^ n (%)		
Northeast	38 (18.0)	34 (18.5)
Midwest	61 (28.9)	56 (30.4)
Southeast	58 (27.5)	39 (21.2)
Southwest	22 (10.4)	18 (9.8)
West	32 (15.2)	37 (20.1)
Urban/suburban/rural, n (%)		
Urban	50 (24.0)	53 (29.3)
Suburban	111 (53.4)	93 (51.4)
Rural	47 (22.6)	35 (19.3)
Not sure	3 (1.4)	3 (1.6)

EoE, eosinophilic esophagitis; GED, General Educational Development; NA, not applicable; SD, standard deviation.

a Response options were not mutually exclusive; participants could select any that applied.

b Other races not included here.

c These included “retired” (n = 1) or other employment status not included here (n = 8), as well as those who preferred not to answer (n = 2).

d These included “less than high school” (n = 5), other education levels not included here (n = 9), as well as those who preferred not to answer (n = 1).

e This is as per the current census.

### Current Symptoms of EoE and Use of Prescription Medications for EoE

Current symptoms of EoE reported by participants are provided in Table A3. The mean (SD) participant-reported time from the first occurrence of symptoms to the time of questionnaire administration was 8.4 (4.6) years in adolescents and 15.6 (11.2) years in adults. The most frequently reported symptoms in adolescents were abdominal pain (30.3%), nausea (28.0%), and regurgitation/reflux (27.0%). In adults, the most frequently reported symptoms were difficulty or discomfort in swallowing solid food (47.3%), avoiding food (42.9%), and heartburn (41.3%). Overall, 15.6% of adolescents and 7.1% of adults reported that they did not have any of the symptoms listed. Although most participants considered the severity of their EoE to be “moderate” (adolescents, 48.3%; adults, 50.5%), 21.3% of adolescents and 23.4% of adults reported that their EoE was “severe” or “very severe”. In addition, the majority of participants were receiving prescription medications to treat their EoE at the time of the survey (adolescents, 83.4%; adults, 72.8%); of these, 66.5% (117/176) of adolescents and 69.4% (93/134) of adults were receiving more than one prescription medication. In total, 63.5% of adolescents and 49.5% of adults also reported that their EoE was moderately controlled (adolescents, 34.1%; adults, 33.7%) or very controlled (adolescents, 29.4%; adults, 15.8%) over the 3 months before they completed the survey.

#### Impact of current symptoms of EoE

Adolescents and adults most frequently considered their current symptoms to have a “mild” or “moderate” impact on their ability to perform daily activities. Over 41% and 34% of adolescents and adults, respectively, were affected moderately in their ability to perform daily activities based on the 3 most frequently reported symptoms. However, at least 11% of adolescents and adults reported that these symptoms were so severe that they were unable to perform daily activities.

### Impact of EoE on Functioning

#### Impact of EoE on vitality

**Figure 1:**
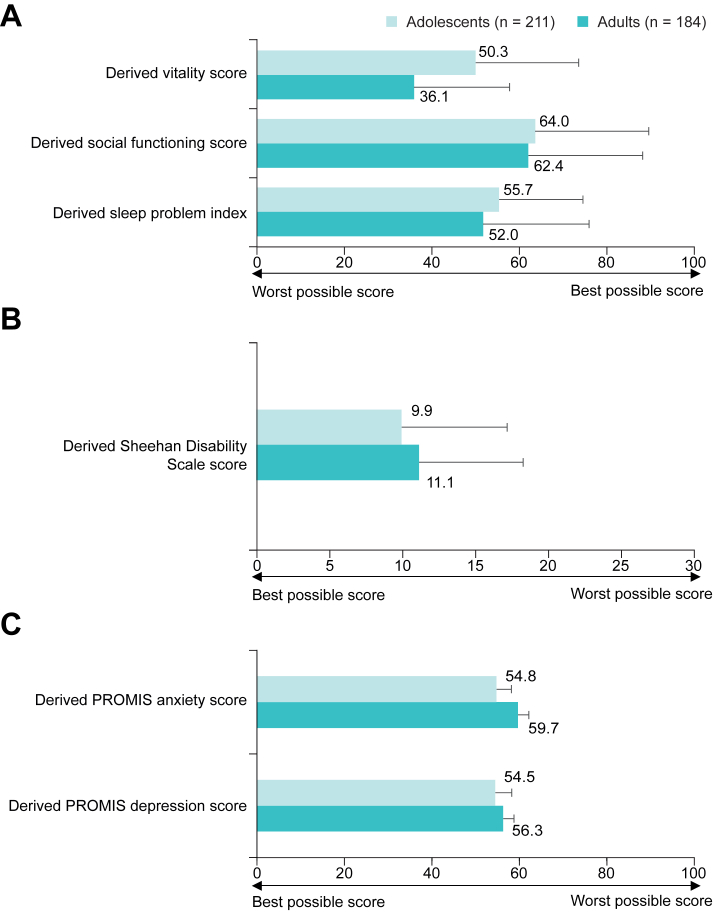
Impact of EoE on (A) the domains of vitality, social functioning, and sleep over the past 4 weeks, calculated as derived scores and (B) school/work, social life/leisure activities and family life/home responsibilities over the past 12 months as measured using the Sheehan Disability Scale scores in adolescents (11–17 years old [caregiver-reported]) and adults (≥18 years old [self-reported]). (C) Derived PROMIS anxiety and depression scores over the past 7 days in adolescents (11–17 years old [caregiver-reported]) and adults (≥18 years old [self-reported]) with EoE. Data are mean (SD). Factors were assessed using the following caregiver- and patient-reported outcome instruments: the impact on sleep was evaluated using 4 questions from the European Health Interview Survey: Developing Common Instruments for Health Surveys^21^; the impact on social functioning and vitality was assessed using 2 items and 4 items from the 36-Item Short Form Health Survey,^19^ respectively; anxiety and depression were assessed using the PROMIS anxiety-8a v1.0 and v2.0, and depression-8b v1.0 and depressive symptoms-6a v2.0 instruments,^24^ respectively; and the impact on school/work, social life/leisure activities and family life/home responsibilities was assessed using the three-item Sheehan Disability Scale.^22^^,^^23^ EoE, eosinophilic esophagitis; PROMIS, Patient-Reported Outcomes Measurement Information System; SD, standard deviation.

Mean (SD) derived SF-36 vitality scores (range, 0–100) were 50.3 (23.7) for adolescents and 36.1 (22.0) for adults, suggesting greater vitality in adolescents than adults with EoE (Figure 1A). In total, 40.3% (85/211) of adolescents and 64.1% (118/184) of adults reported feeling worn out “all of the time”, “most of the time”, or “a good bit of the time”. The most frequently reported response to “did you have a lot of energy” was “some of the time” for adolescents (28.4% [60/211]) and “a little of the time” for adults (28.8% [53/184]). Similarly, 40.3% (85/211) of adolescents and 64.7% (119/184) of adults reported feeling tired “all of the time”, “most of the time”, or “a good bit of the time”.

#### Impact of EoE on social functioning

Mean (SD) derived SF-36 social functioning scores (range, 0–100) were similar for adolescents (64.0 [26.1]) and adults (62.4 [26.3]), suggesting that participants experienced a moderate EoE-associated impact on social functioning (Figure 1A). Adolescents and adults reported that the extent of interference with their normal social activities was “quite a bit” (10.9% [23/211] and 20.7% [38/184], respectively), and that they experienced this interference “a good bit of the time” (38.4% [81/211] and 32.6% [60/184], respectively). Notably, no participants answered “none of the time” when asked how much of the time their symptoms of EoE interfered with their normal social activities.

#### Impact of EoE on sleep

Mean (SD) derived sleep problem indices (range, 0–100) suggested moderate sleep problems in adolescents and adults (55.7 [19.3] and 52.0 [24.4], respectively) (Figure 1A). Overall, 50.2% (106/211) of adolescents and 62.5% (115/184) of adults reported problems falling or staying asleep owing to EoE. Of these, 60.4% (64/106) of adolescents and 57.4% (66/115) of adults reported problems falling asleep “all of the time”, “most of the time”, or “a good bit of the time”. The proportions of adolescents and adults who reported waking up frequently during the night “all of the time”, “most of the time”, or “a good bit of the time” were 54.7% (58/106) and 67.0% (77/115), respectively.

#### Impact of EoE on impairment

Mean (SD) derived SDS scores (range, 0–30) suggested a slightly lower impact of EoE on impairment in adolescents (9.9 [7.2]) than adults (11.1 [7.1]) (Figure 1B). Most adolescents and adults (81.2% [168/207] and 55.9% [99/177], respectively) reported missing school/work over the past 12 months owing to EoE. Similar proportions of adolescents (22.7%) and adults (22.8%) reported impairment at school/work, whereas fewer adolescents than adults reported disruption to social life/leisure activities (20.4% and 26.6%, respectively) and family life/home responsibilities (18.5% and 32.1%, respectively) (Figure A2).

#### Current symptoms of anxiety and depression

In adolescents and adults, standardized means (SD) for the derived PROMIS anxiety scores were 54.8 (3.4) and 59.7 (2.5), respectively; scores were higher than 50, indicating that participants experienced more symptoms of anxiety than the general population (Figure 1C). In the PROMIS anxiety-8a v2.0 form, caregivers reported that their child felt “worried” and “nervous” over the past 7 days “sometimes” (34.1% [72/211] and 37.4% [79/211], respectively), “often” (17.5% [37/211] and 16.6% [35/211], respectively), or “almost always” (6.2% [13/211] and 7.1% [15/211], respectively). In the PROMIS anxiety-8a v1.0 form, adults reported feeling “anxious” and “nervous” over the past 7 days “sometimes” (28.8% [53/184] and 33.7% [62/184], respectively), “often” (26.1% [48/184] and 19.0% [35/184], respectively), or “always” (8.7% [16/184] and 8.2% [15/184], respectively).

Standardized means (SD) for the derived PROMIS depression scores were 54.5 (3.8) and 56.3 (2.5) in adolescents and adults, respectively; scores were higher than 50, indicating that adolescents and adults with EoE experienced more symptoms of depression than the general population (Figure 1C). In the PROMIS depressive symptoms-6a v2.0 form, caregivers reported that their child felt “sad” and “lonely” over the past 7 days “sometimes” (34.1% [72/211] and 27.5% [58/211], respectively), “often” (11.4% [24/211] and 19.0% [40/211], respectively), or “almost always” (1.9% [4/211] and 4.7% [10/211], respectively). Approximately one-third of caregivers of adolescents reported being “very bothered” for each depression symptom; caregivers most frequently reported being “very bothered” at times when their child felt “lonely” (37.0%), demonstrating that in children with EoE, the whole family may also be affected. In the PROMIS depression-8b v1.0 form, adults reported feeling “depressed” and “sad” over the past 7 days “sometimes” (27.7% [51/184] and 35.3% [65/184], respectively), “often” (13.6% [25/184] and 19.6% [36/184], respectively), or “always” (4.3% [8/184] and 5.4% [10/184], respectively).

### Perceived Stigma

The majority of participants reported experiencing stigma over the past year owing to EoE (adolescents: 59.7% [126/211]; adults: 57.6% [106/184]; Table 2). The most common sources of stigma were family (47.6% [60/126]), friends (44.4% [56/126]), and classmates (41.3% [52/126]) in adolescents, and family (73.6% [78/106]), friends (63.2% [67/106]), and HCPs (36.8% [39/106]) in adults (Table 2).

**Table 2:** Proportions of Adolescents (11–17 Years Old [Caregiver-Reported]) and Adults (≥18 Years Old [Self-Reported]) With EoE Who Reported Experiencing Stigma, and the Reported Sources of Perceived Stigma, Over the Past 12 Months

Category, n (%)	Adolescents (n = 211)	Adults (n = 184)
Over the past year, have you [has your child] ever experienced any stigma from anyone (eg friends, family members, coworkers, classmates) due to your [his/her] EoE symptoms?		
Yes	126 (59.7)	106 (57.6)
No	68 (32.2)	71 (38.6)
I am not sure	17 (8.1)	7 (3.8)
Over the past year, with whom have you [has your child] experienced any type of stigma due to your [his/her] EoE symptoms?^a^^,^^b^		
My family (eg child, spouse, brother, sister, parents, cousins, grandparents)	60 (47.6)	78 (73.6)
My friends	56 (44.4)	67 (63.2)
My health-care providers	28 (22.2)	39 (36.8)
My coworkers	NA	30 (28.3)
My boss/supervisor or employer	NA	20 (18.9)
My classmates	52 (41.3)	NA
My teacher	37 (29.4)	NA
Other person(s)	16 (12.7)	NA

EoE, eosinophilic esophagitis; NA, not applicable.

a Including participants who responded “Yes” to experiencing any stigma (adolescents, n = 126; adults, n = 106).

b Participants could select all responses that applied.

#### Impact of perceived stigma

**Figure 2:**
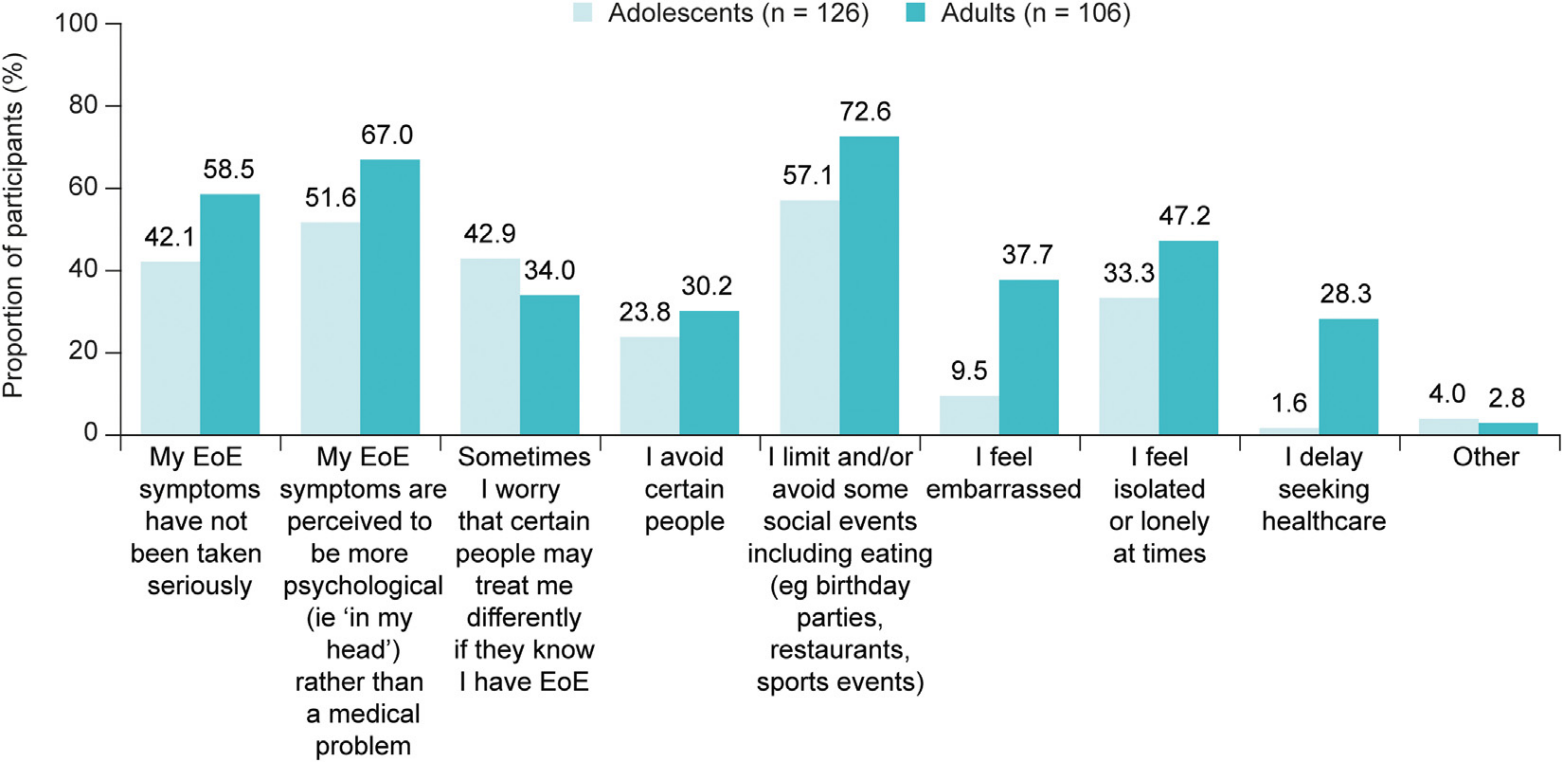
Summary of the impact of perceived stigma over the past 12 months in adolescents (11–17 years old [caregiver-reported]) and adults (≥18 years old [self-reported]) with EoE. In this study, social stigma was defined as a participant feeling discriminated against or viewed negatively by other people because of their EoE symptoms. Results are from participants who responded “Yes” (adolescents, n = 126; adults, n = 106) to experiencing any stigma; participants who reported “I am not sure” are not included. Participants could select all responses that applied. EoE, eosinophilic esophagitis.

Among participants who reported experiencing stigma, the reported impact of this was generally lower for adolescents than adults (Figure 2). The impact of perceived stigma in adolescents and adults included: “I limit and/or avoid some social events including eating (eg birthday parties, restaurants, sports events)” (57.1% [72/126] and 72.6% [77/106], respectively), “my EoE symptoms are perceived to be more psychological (ie ‘in my head’) rather than a medical problem” (51.6% [65/126] and 67.0% [71/106], respectively), and “my symptoms have not been taken seriously” (42.1% [53/126] and 58.5% [62/106], respectively). Substantially more adults than adolescents reported a resultant delay in seeking health care (adolescents, 1.6% [2/126]; adults, 28.3% [30/106]).

## Discussion

This noninterventional, cross-sectional, web-based survey assessed the impact of EoE on the daily life and ability to function, and perceived stigma in adolescents and adults with EoE in the United States of America. This study demonstrated that EoE has a substantial effect on vitality, social functioning, sleep, and the daily life of patients regardless of age. Symptoms of anxiety and depression were more common in patients with EoE compared with the general population. The majority of participants experienced EoE-associated stigma, most commonly from relatives, friends, classmates, or HCPs. The symptoms of EoE most frequently reported during our study were generally consistent with those described elsewhere,^25^, ^26^, ^27^ indicating that the participants in this study were representative of the general population with EoE in this regard. Our findings also support the well-established hypothesis that symptoms of EoE can substantially impair patients’ HRQoL, including negatively affecting vitality, social life, and sleep.^2^^,^^11^

Evidence indicates that patients with EoE with sleep disturbances,^28^ psychological concerns,^14^ or perceived stigma^15^ have poor HRQoL; a negative correlation between HRQoL and the severity of patient-reported symptoms or the duration of the disease has also been observed in EoE.^29^, ^30^, ^31^ In our study, approximately 20% of participants perceived their symptoms to be severe or very severe, and the mean time since diagnosis was approximately 6 years. Histology was not examined during our study, and therefore it is challenging to determine the full extent of the disease activity in our population, particularly because symptoms alone are generally considered unreliable for determining disease activity in EoE.^32^ Although our study did not correlate HRQoL with symptoms or disease severity directly, it is possible that these factors contributed to the poor HRQoL in the adolescent and adult participants.

In our study, adults with EoE, despite 72.8% receiving treatment with prescription medications, had worse vitality and social functioning, as measured using the SF-36 (36.1 and 62.4, respectively) than healthy adults (∼75 and ∼90, respectively),^33^ adults with ulcerative colitis (UC) in remission (62.0 and 86.1, respectively),^34^ patients with Crohn’s disease (51 and 81, respectively),^35^ and adults with asthma and rhinitis (62.4 and 88.5, respectively), most of whom were also receiving treatment for their disease. In another adult population with EoE who had received topical corticosteroids for 2 months, mean SF-36 scores for vitality were the same at baseline and after treatment (67.0 and 67.0, respectively), whereas mean SF-36 scores for social functioning improved slightly from baseline to after treatment (82.0 and 90.0, respectively).^36^ Adults with EoE in our study had a longer mean time since diagnosis (∼6 years) compared with the other study in EoE (≥1 year),^36^ which may help to explain these findings, because HRQoL can decrease with a longer duration of EoE.^30^ Conversely, our findings were similar to those reported for a patient population with IBD who received active medical intervention for 6 months (mean SF-36 scores: vitality, 26.6; social functioning, 62.6).^37^ In terms of sleep health, in our study, 50.2% of adolescents and 62.5% of adults reported problems falling or staying asleep, which aligns with another study in EoE that observed difficulty sleeping/insomnia in 42.9% and 50.0% of adolescents and adults, respectively.^2^ Overall, our results, together with the findings from other studies, indicate that patients with EoE experience an impact on their HRQoL, especially in the areas of vitality, social functioning, and sleep. Our findings may also suggest that patients with EoE have similar or sometimes worse HRQoL when compared with patients with other gastrointestinal diseases or allergic conditions; however, further studies are required to corroborate this observation.

Managing dietary limitations, coping strategies, and symptoms of food impaction or dysphagia can contribute substantially to psychosocial and psychological difficulties in young patients, particularly because social activities are often centered around eating.^12^^,^^38^ In a study of adults with EoE, 65% reported experiencing social embarrassment or distress, which was often associated with having a choking episode at social occasions (38%) or causing others concern (21%).^13^ The psychosocial and psychological concerns in patients with EoE appear to be related mainly to symptoms and disease management strategies.

In our study, 22.7% of adolescents had EoE-associated impairment of school/work, which was similar to the proportion of adolescents (28.6%) who experienced difficulties completing their daily activities at home, work, or school in another study of EoE.^2^ Studies in other chronic diseases suggest that approximately 20%–30% of adults experience work disability (Crohn’s disease: 29%; UC: 19%) compared with an age-adjusted general population (7%).^39^ The factors associated with work disability in patients with Crohn’s disease were female sex, severe disease, and disease duration,^39^ which also aligned with some of the characteristics of the adult population in our study. Our findings further support the consensus that EoE has a negative impact on school or work, and the proportion of patients experiencing disruption to their work tends to be similar to other gastrointestinal conditions, such as Crohn’s disease.

Fear of potential side effects, adherence to a restrictive dietary regimen, concerns about symptoms, comorbid food allergies, social embarrassment, school avoidance, feeding difficulties, persistent pain, and sleep disturbances can affect the emotional and psychological well-being of patients with EoE.^12^^,^^14^ In a retrospective study in EoE, at least one in 7 children and one in 3 adults had psychiatric comorbidities, of which anxiety (23%) and depression (17%) were the most common.^40^ Consistent with previous findings,^40^ mean PROMIS scores for anxiety and depression in our study in adolescents (54.8 and 54.5, respectively) and adults (59.7 and 56.3, respectively) also indicated greater depression and anxiety when compared with the general population (score of 50 for each domain). In our study, adolescents with EoE experienced slightly greater anxiety and depression than adolescents with Crohn’s disease (mean PROMIS pediatric T-scores of 46.9 and 43.2, respectively)^41^ or UC (mean PROMIS pediatric T-scores of 50.1 and 46.2).^42^ Mean PROMIS scores were slightly lower for anxiety and depression in adults with Crohn’s disease (56.4 and 52.4, respectively) or UC (54.5 and 50.0) than in participants in our study.^43^ In our study, depression and anxiety were more pronounced for adults than adolescents, which is consistent with reports that suggest depression and anxiety can increase with age in EoE.^44^ In addition, the adult population in our study comprised more women than men, and the mean age was 35.5 years; this population is considered at greater risk of mental distress than men or patients in other age groups.^45^ In line with other studies, our findings also indicated that patients with EoE are prone to anxiety and depressive symptoms, and the level of these psychological concerns seems to be similar to other conditions, such as Crohn’s disease and UC.

Perceived stigma is well documented in conditions including human immunodeficiency virus/acquired immunodeficiency syndrome,^46^ mental illness,^47^ and cancer.^48^ The impact of perceived stigma varies from physical limitations^49^ to depression^46^^,^^48^ and suicidal ideation,^46^ as reported by studies in refractory epilepsy,^49^ cancer,^48^ and human immunodeficiency virus/acquired immunodeficiency syndrome.^46^ Prolonged perceived stigma may cause increased stress, for which the subsequent increased cortisol levels can lead to negative health outcomes.^50^ In our study, more than half of participants reported experiencing perceived stigma, and this was similar between adolescents and adults (59.7% vs 57.6%). In adolescents, other studies have shown that social stigma associated with dietary management is common with celiac disease^51^ and food allergies^52^ and is also experienced by adolescents with EoE at family and social events.^16^^,^^38^ In adults, a higher proportion of patients with other chronic conditions, such as asthma (78%)^53^ and IBD (84%),^17^ reported experiencing perceived stigma compared with our findings (57.6%).

In our study, the most common sources of stigma were relatives and friends; most participants reported limiting or avoiding of social events that involved eating or physical activities as a result. Over one-third of adults (36.8%) reported experiencing stigma from HCPs, and some (28.3%) delayed seeking health care because of the stigma experienced. This aligns with previous findings in patients with IBD, suggesting that health-seeking behaviors and relationships with HCPs can be negatively influenced by perceptions of stigma.^54^ For patients with EoE, a prolonged duration of untreated disease after diagnostic delay can lead to an increased prevalence of esophageal strictures, potentially worsening psychological outcomes,^55^ highlighting a potential risk of health-care avoidance. Although patients in our study reported experiencing perceived stigma, these reports were lower than those seen in other gastrointestinal diseases; however, patients with EoE who delay seeking health care owing to stigma may experience detrimental effects on their health as a result. Encouraging treatment-seeking behavior and raising public awareness of EoE may help to address this issue.

Limitations of this study include the potential responder bias due to the study design and the use of generic survey instruments that are not validated or designed specifically for use in EoE; however, the use of generic survey instruments enabled valuable comparison with other diseases and the general population. Recruiting participants from a patient advocacy group may represent a more engaged population than the general public which, with convenience sampling and the gift card incentive, could have led to either an underestimation or overestimation of the results. The survey could only be completed online, limiting participation to patients with internet access. Moreover, the study findings were assessed descriptively; future studies involving statistical testing may provide further insights into the associations between various HRQoL parameters in patients with EoE. The patient-reported outcomes may not reflect the experiences of racial minorities because the population in our study predominantly identified as White. Further studies on this underrepresented demographic would not only be informative but could improve health outcomes, considering ethnic/racial minorities tend to have poor HRQoL and heightened stigma perceptions.^56^^,^^57^ The study population also comprised mostly women, and because EoE is reported to predominantly affect men,^58^ our results may not be fully generalizable to the broader EoE patient population. The physician diagnosis of EoE and other clinical outcomes, including symptoms of EoE, were caregiver- or self-reported, and so could not be corroborated by medical records/physician reports. Furthermore, caregiver responses may not reflect adolescents’ true experiences. Although the survey determined whether participants were taking prescription medications for EoE, there were no questions in place to capture the type of treatments used or the subsequent treatment outcomes. It was also not possible to confirm that experiences of anxiety and depression were associated with EoE.

This study was conducted in a large sample size of adolescents and adults, recruited from 5 different census regions in the United States of America, and provides real-world insights into the humanistic burden of EoE. Generic tools were used to capture outcomes related to HRQoL and perceived stigma, which enabled robust comparisons with other disease states, as well as the general population.^19^, ^20^, ^21^, ^22^, ^23^, ^24^ The recruitment process with limited inclusion or exclusion criteria ensured participation from a heterogeneous population with EoE.

## Conclusion

In summary, we found that EoE substantially affects patients’ vitality, social functioning, and sleep, which can lead to anxiety and depression, as well as perceived stigma. Although functional status and disease severity in EoE may not be considered as severe as in other gastrointestinal conditions,^59^ patients with these diseases appear to experience similar social and emotional consequences. It is, therefore, important to understand the impact of EoE on patients’ lives to tailor treatment plans that help to maximize treatment benefits and improve patients’ HRQoL. The use of multidisciplinary teams and psychological support for patients with EoE should be considered.
