# Supplementary material for: Health-Related Quality of Life and Perceived Stigma in Eosinophilic Esophagitis: A Real-World, US, Web-Based Survey
Source: Gastro Hep Adv. 2024 Jul 30;3(8):1087–97. doi: 10.1016/j.gastha.2024.07.015 (PMC11550744; doi:10.1016/j.gastha.2024.07.015)
Supplement: Supplementary Materials [file mmc1.docx]

**SUPPLEMENTARY INFORMATION**

**
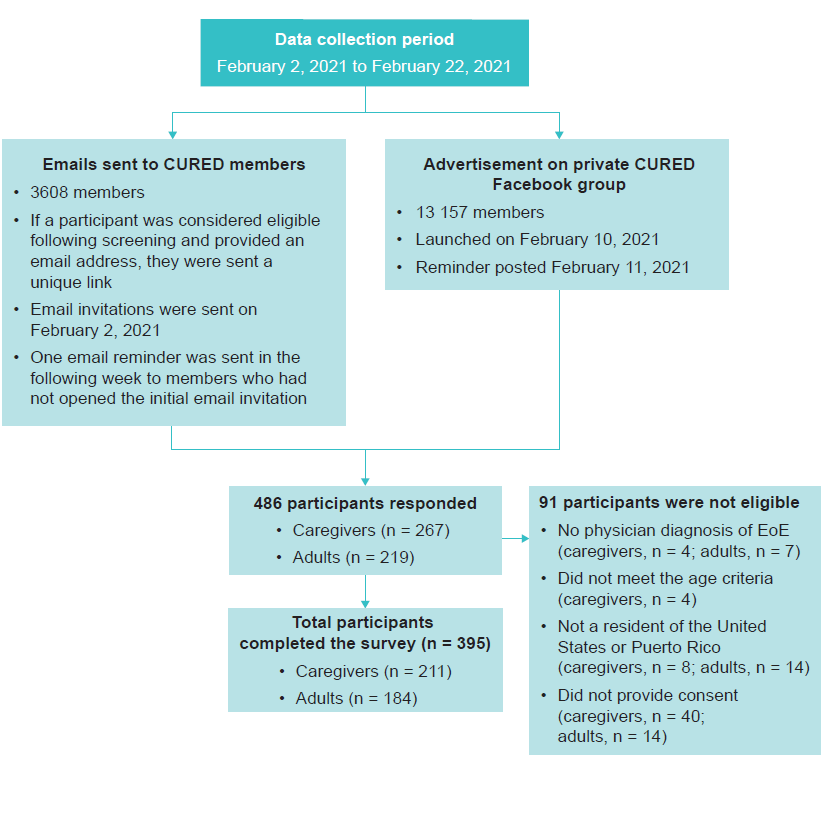
**

**Figure A1.** Participant enrollment diagram. CURED, Campaign Urging Research for Eosinophilic Disease; EoE, eosinophilic esophagitis.

**Table A1.** Summary Scoring Used to Assess the Impact of EoE on the Domains of Vitality and Social Functioning in Adolescents (11–17 Years Old [Caregiver-Reported]) and Adults (≥18 Years Old [Self-Reported])

| **Items** | **Original response score** | **Recoded value** |
| --- | --- | --- |
| *Vitality^a^* | | |
| SF-36 item numbers 23 and 27 | 1 | 100 |
|  | 2 | 80 |
|  | 3 | 60 |
|  | 4 | 40 |
|  | 5 | 20 |
|  | 6 | 0 |
| SF-36 item numbers 29 and 31 | 1 | 0 |
|  | 2 | 20 |
|  | 3 | 40 |
|  | 4 | 60 |
|  | 5 | 80 |
|  | 6 | 100 |
| *Social Functioning^b^* | | |
| SF-36 item number 20 | 1 | 100 |
|  | 2 | 75 |
|  | 3 | 50 |
|  | 4 | 25 |
|  | 5 | 0 |
| SF-36 item number 32 | 1 | 0 |
|  | 2 | 25 |
|  | 3 | 50 |
|  | 4 | 75 |
|  | 5 | 100 |

EoE, eosinophilic esophagitis; RAND, Research and Development.

^a^Participants were asked to score each of the 4 vitality questions from “all of the time” (score 1) to "none of the time” (score 6).^1^ These scores were transformed into summary scores ranging from 0 to 100 using the RAND Health, 2017 scoring algorithm, with higher scores indicating greater vitality.^2^ Summary scores were only calculated for participants who responded to over 50% of questions. After recoding, scores were averaged to give a total range for each domain of 0 to 100.

^b^Participants were asked to score each of the 2 questions related to social functioning from “not at all” (score 1) to “extremely” (score 5) or “all of the time” (score 1) to “none of the time” (score 6).^1^ These scores were transformed into summary scores ranging from 0 to 100 using the RAND Health, 2017 scoring algorithm, with higher scores indicating fewer limitations on social functioning (**Table A2**).^2^ Scores were considered to be missing if one of the 2 items was not completed for a participant. After recoding, scores were averaged to give a total range for each domain of 0–100.

**Table A2.** Questionnaire Used to Assess Perceptions of Stigma Associated With EoE in Adolescents (11–17 Years Old [Caregiver-Reported]) and Adults (≥18 Years old [Self-Reported])

| **Question** | **Response option** |
| --- | --- |
| Over the past year, have you [has your child] ever experienced any type of stigma due to your [his/her] EoE symptoms? | Yes |
|  | No |
|  | I am not sure |
| Over the past year, with whom have you [has your child] experienced any type of stigma due to your [his/her] EoE symptoms? Select all that apply. | My [my child’s] family (e.g. child, spouse, brother, sister, parents, cousins, grandparents) |
|  | My [my child’s] friends |
|  | My [my child’s] healthcare providers |
|  | My [my child’s] coworkers |
|  | My boss/supervisor or employer |
|  | My [my child’s] classmates |
|  | My [my child’s] teacher |
|  | Other person(s) |
| Over the past year, what has been the impact on you [your child] as a result of experiencing the stigma of EoE symptoms? Select all that apply. | My [my child’s] EoE symptoms have not been taken seriously |
|  | My [my child’s] EoE symptoms are perceived to be more psychological (i.e. 'in my head') rather than a medical problem |
|  | Sometimes I worry [my child worries] that certain people may treat me [my child] differently if they know I have [my child has] EoE |
|  | I avoid [my child avoids] certain people |
|  | I limit and/or avoid [my child limits and/or avoids] some social events including eating (e.g., birthday parties, restaurants, sports events) |
|  | I feel [my child feels] embarrassed |
|  | I feel [my child feels] isolated or lonely at times |
|  | I delay [my child delays] seeking health care |
|  | Other |

EoE, eosinophilic esophagitis.

**Table A3.** Current Symptoms of EoE Reported by Adolescents (11–17 Years Old [Caregiver-Reported]) and Adults (≥18 Years Old [Self-Reported]).

| **Current symptoms of EoE, n (%)** | **Adolescents**  **(n = 211)** | **Adults**  **(n = 184)** |
| --- | --- | --- |
| Abdominal pain | 64 (30.3) | 65 (35.3) |
| Nausea | 59 (28.0) | 54 (29.3) |
| Regurgitation/reflux | 57 (27.0) | 56 (30.4) |
| Avoiding food | 56 (26.5) | 79 (42.9) |
| Heartburn | 56 (26.5) | 76 (41.3) |
| Difficulty or discomfort in swallowing solid food | 49 (23.2) | 87 (47.3) |
| Poor weight gain/growth | 36 (17.1) | 16 (8.7) |
| Feeling of an obstruction in the throat | 35 (16.6) | 51 (27.7) |
| Decrease in appetite | 35 (16.6) | 36 (19.6) |
| Vomiting | 34 (16.1) | 35 (19.0) |
| I currently do not have any of these symptoms | 33 (15.6) | 13 (7.1) |
| Chest pain | 28 (13.3) | 57 (31.0) |
| Diarrhea | 27 (12.8) | 35 (19.0) |
| Food impaction/obstruction | 16 (7.6) | 39 (21.2) |
| Other | 14 (6.6) | 9 (4.9) |
| Esophageal perforation(s) | 10 (4.7) | 6 (3.3) |
| Weight loss | 9 (4.3) | 9 (4.9) |
| Difficulty or discomfort in swallowing liquids | 6 (2.8) | 37 (20.1) |

EoE, eosinophilic esophagitis.

Participants could select more than one response. There was an open-ended response option to allow participants to report any symptoms not listed. Sample size is provided for the verbatim responses reported more than once. The symptoms reported verbatim by caregivers of adolescents were: asthma; cannot gain weight; chronic constipation (n = 3); chronic cough; coughing when eating; eczema; “he has a GI-continuous feeds. Little by mouth”; increase in appetite due to steroid to control; increase in phlegm/congestion; loose stools but not diarrhea; no symptoms while taking prevacid; rashes, anxiety, reflux, random dry heaving; and shuts off formula feedings. The symptoms reported verbatim by adults were: burning; burping, feeling of air bubble in throat/chest; esophagus spasms; excessive salivating and water brash; feeling of mucus in throat; “I use a feeding tube for formula”; sore throat, scratchy feeling and hoarse voice; swelling, inability to lose weight; and weird feeling/tingling in throat.


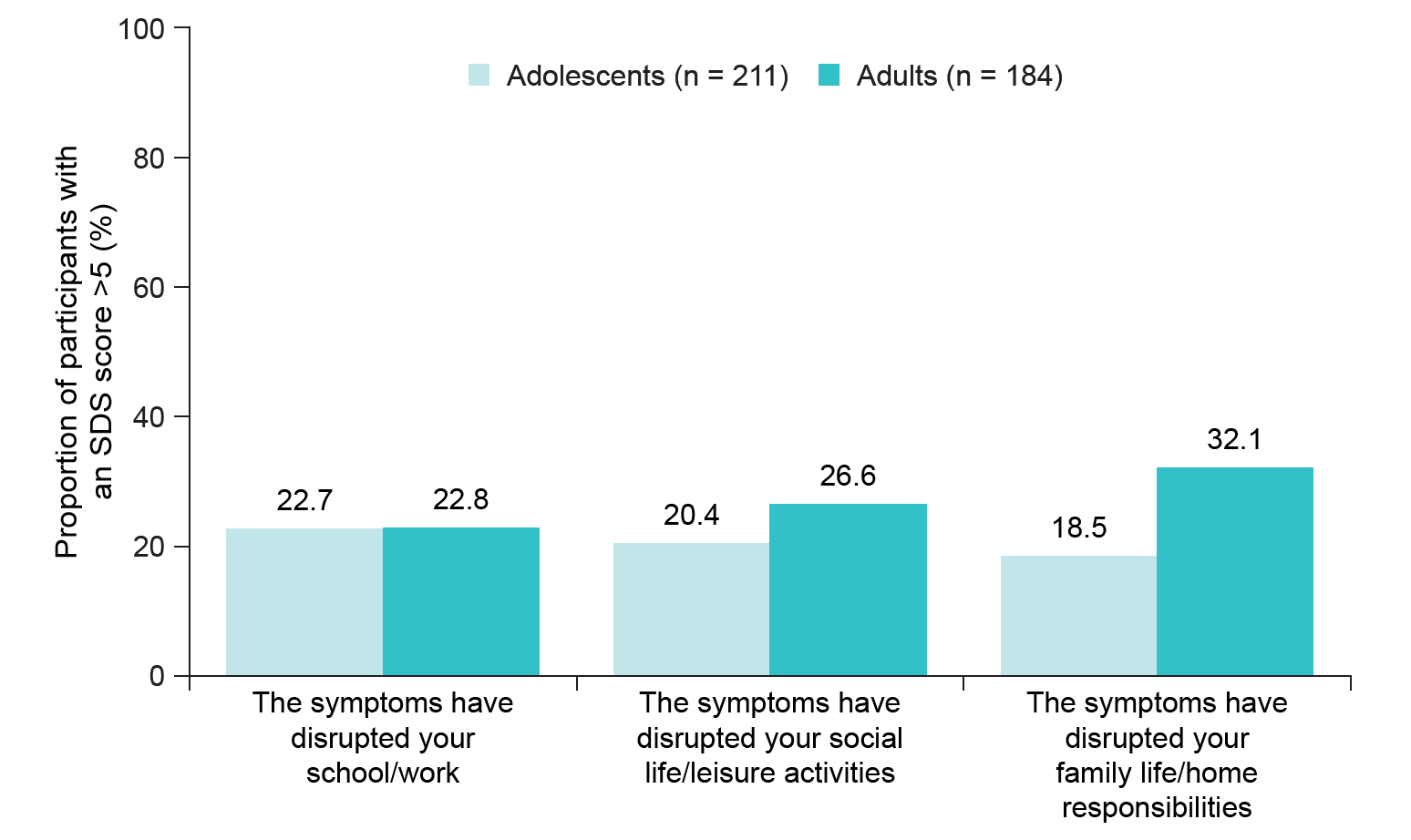


**Figure A2.** Impact of EoE on school/work, social life/leisure activities, and family life/home responsibilities over the past 12 months. The impairment due to EoE in each of the 3 areas was assessed using the SDS,^3,4^ with scores ranging from 0 (not at all) to 10 (extremely), where scores greater than 5 were suggestive of impairment in that area. In total, 1.4% of adolescents (n = 3) and 17.4% of adults (n = 32) reported that they had not worked/studied at all during the past 4 weeks for reasons that were unrelated to EoE. EoE, eosinophilic esophagitis; SDS, Sheehan Disability Scale.

**References**

1. Hays RD, Sherbourne CD, Mazel RM. The RAND 36-Item Health Survey 1.0. Health Econ 1993;2:217–227.

2. RAND Health. 36-Item Short Form Survey (SF-36) Scoring Instructions. <http://www.rand.org/health/surveys_tools/mos/36-item-short-form/scoring.html>. Accessed October 18, 2023.

3. Sheehan DV. The anxiety disease. New York, NY: Scribner, 1983.

4. Williams, JBW. Mental health status, functioning, and disabilities measures. In: American Psychiatric Association Task Force. Task Force for the Handbook of Psychiatric Measures. Handbook of psychiatric measures. Washington, DC: American Psychiatric Association, 2000, 93–115.
